# Supplementary material for: First-Trimester Screening for Fetal Growth Restriction and Small-for-Gestational-Age Pregnancies without Preeclampsia Using Cardiovascular Disease-Associated MicroRNA Biomarkers
Source: Biomedicines. 2022 Mar 19;10(3):718. doi: 10.3390/biomedicines10030718 (PMC8945808; doi:10.3390/biomedicines10030718)
Supplement: Supplementary file 1 [file biomedicines-10-00718-s001.zip › Supplementary Figure S1.pdf]

Supplementary Figure S1.

A

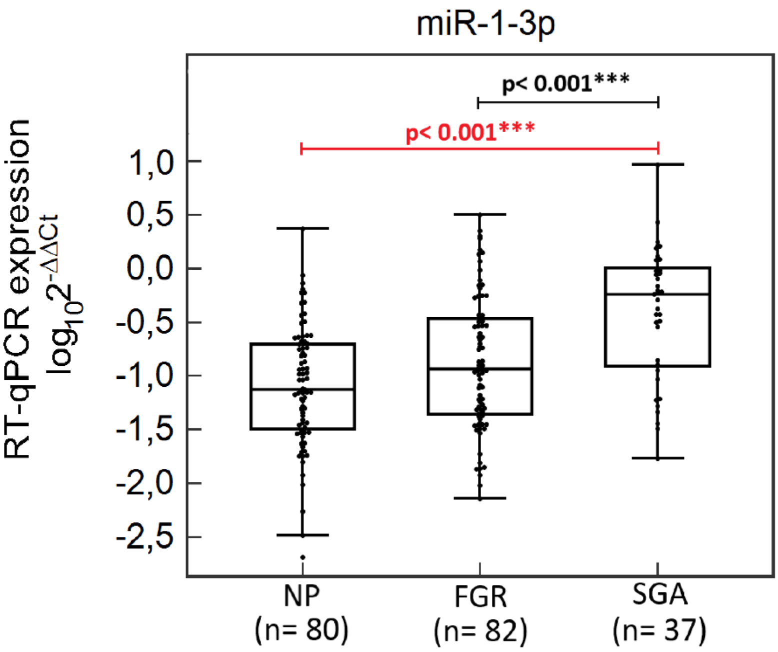

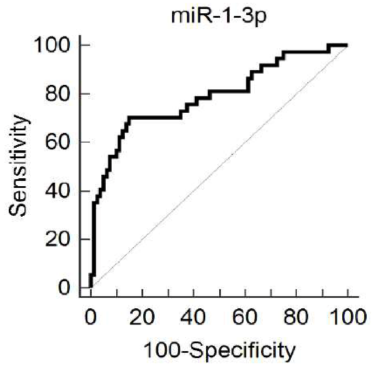

Area under the ROC curve (AUC)	0.790
Standard Error	0.0489
95% Confidence interval	0.705 to 0.860
Significance level P (Area=0.5)	<0.0001

Estimated sensitivity at fixed specificity			
Specificity	Sensitivity	95% CI	Criterion
90.00	56.76	32.43 to 75.68	>0.377841

Criterion	Sensitivity	95% CI	Specificity	95% CI	+LR	95% CI	-LR	95% CI
>0.23812	70.27	53.0 - 84.1	85.00	75.3 - 92.0	4.68	2.7 - 8.2	0.35	0.2 - 0.6

B

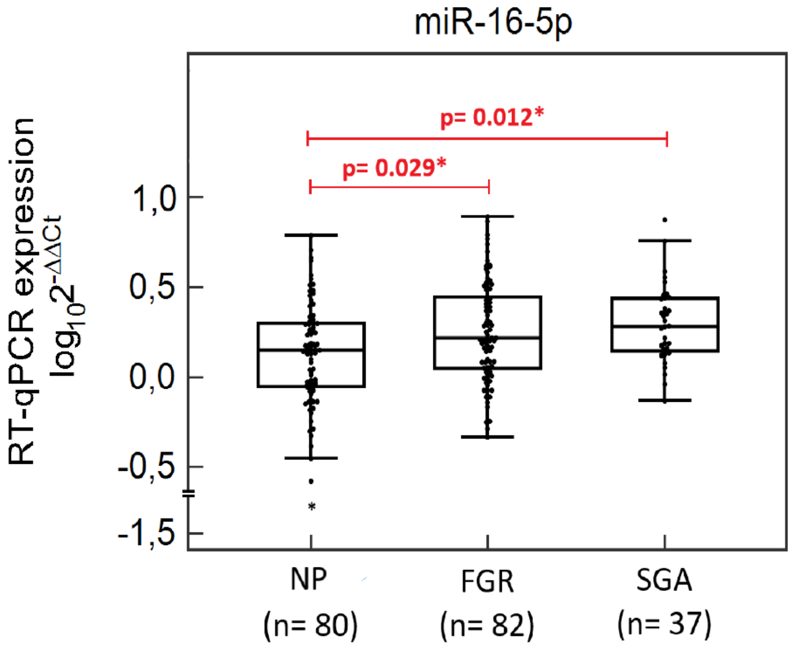

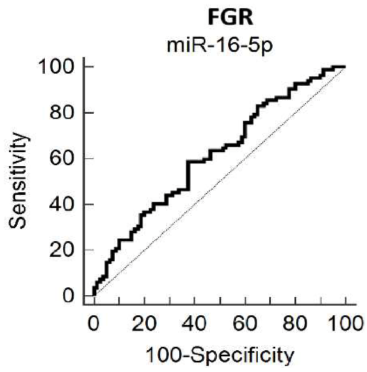

Area under the ROC curve (AUC)	0.615
Standard Error	0.0440
95% Confidence interval	0.536 to 0.690
Significance level P (Area=0.5)	0.0090

Estimated sensitivity at fixed specificity			
Specificity	Sensitivity	95% CI	Criterion
90.00	24.39	12.20 to 39.79	>3.02212

Criterion	Sensitivity	95% CI	Specificity	95% CI	+LR	95% CI	-LR	95% CI
>1.545201	58.54	47.1 - 69.3	62.50	51.0 - 73.1	1.56	1.1 - 2.2	0.66	0.5 - 0.9

C

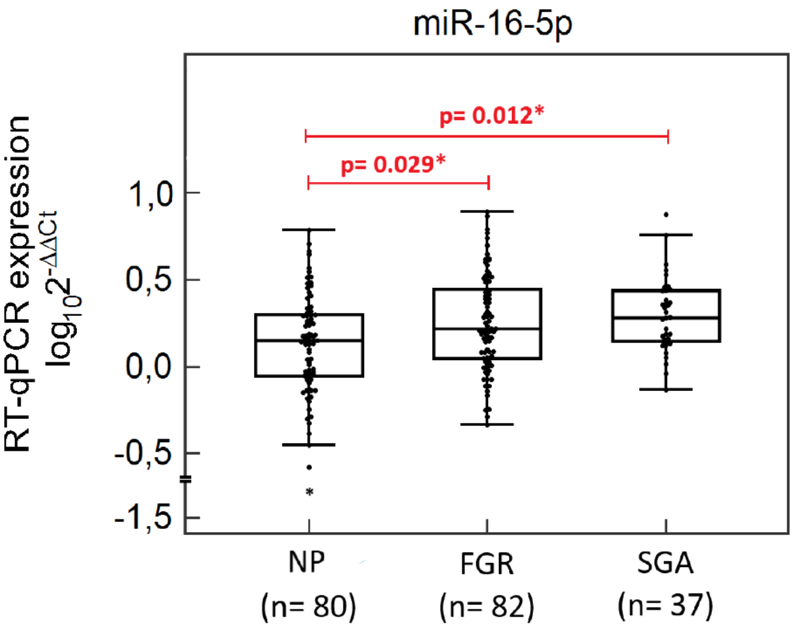

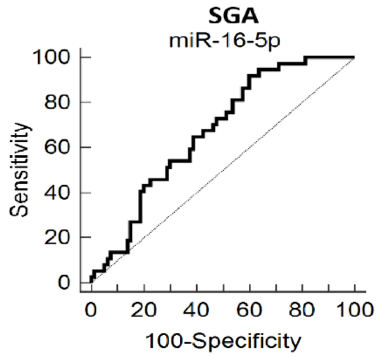

Area under the ROC curve (AUC)	0.672
Standard Error	0.0501
95% Confidence interval	0.579 to 0.756
Significance level P (Area=0.5)	0.0006

Estimated sensitivity at fixed specificity			
Specificity	Sensitivity	95% CI	Criterion
90.00	13.51	2.70 to 43.23	>3.02212

Criterion	Sensitivity	95% CI	Specificity	95% CI	+LR	95% CI	-LR	95% CI
>1.103306	91.89	78.1 - 98.3	40.00	29.2 - 51.6	1.53	1.3 - 1.9	0.20	0.07 - 0.6

D

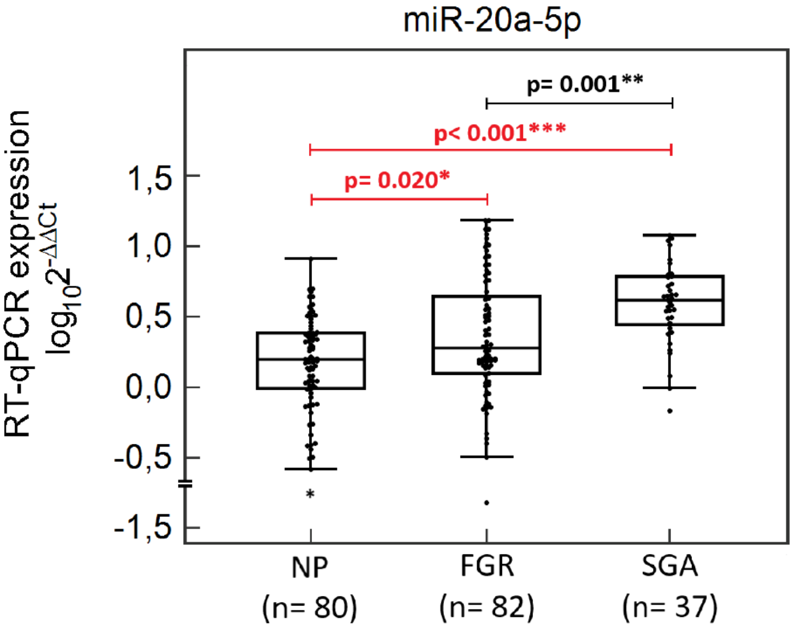

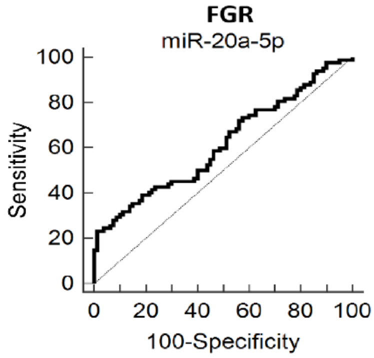

Area under the ROC curve (AUC)	0.615
Standard Error	0.0442
95% Confidence interval	0.535 to 0.690
Significance level P (Area=0.5)	0.0094

Estimated sensitivity at fixed specificity			
Specificity	Sensitivity	95% CI	Criterion
90.00	30.49	18.29 to 42.68	>3.587078

Criterion	Sensitivity	95% CI	Specificity	95% CI	+LR	95% CI	-LR	95% CI
>5.003977	23.17	14.6 - 33.8	98.75	93.2 - 100.0	18.54	2.5 - 135.2	0.78	0.7 - 0.9

G

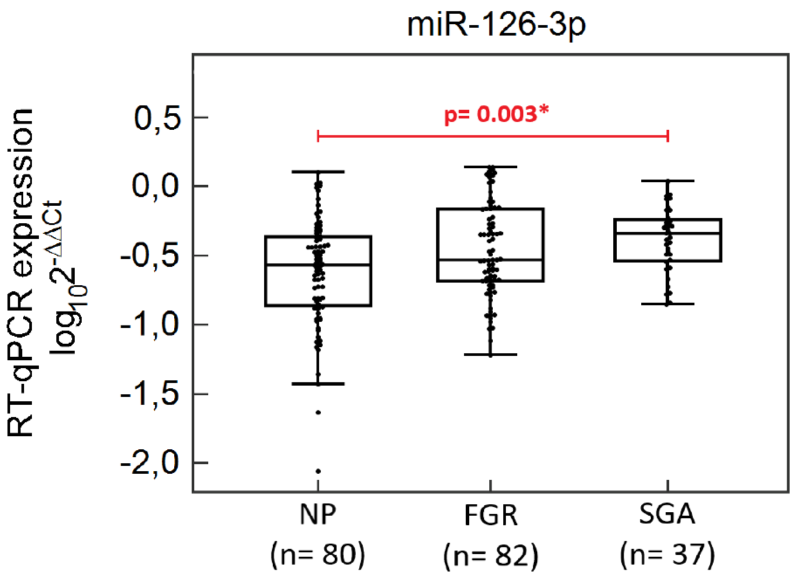

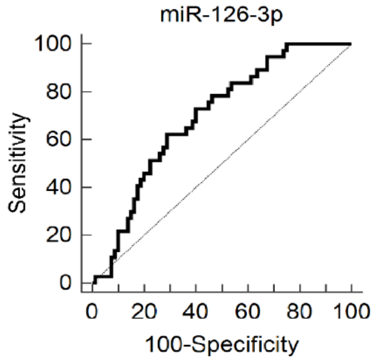

Area under the ROC curve (AUC)	0.695
Standard Error	0.0494
95% Confidence interval	0.603 to 0.777
Significance level P (Area=0.5)	0.0001

Estimated sensitivity at fixed specificity			
Specificity	Sensitivity	95% CI	Criterion
90.00	21.62	2.70 to 56.76	>0.659038

Criterion	Sensitivity	95% CI	Specificity	95% CI	+LR	95% CI	-LR	95% CI
>0.376441	62.16	44.8 - 77.5	71.25	60.0 - 80.8	2.16	1.4 - 3.3	0.53	0.3 - 0.8

H

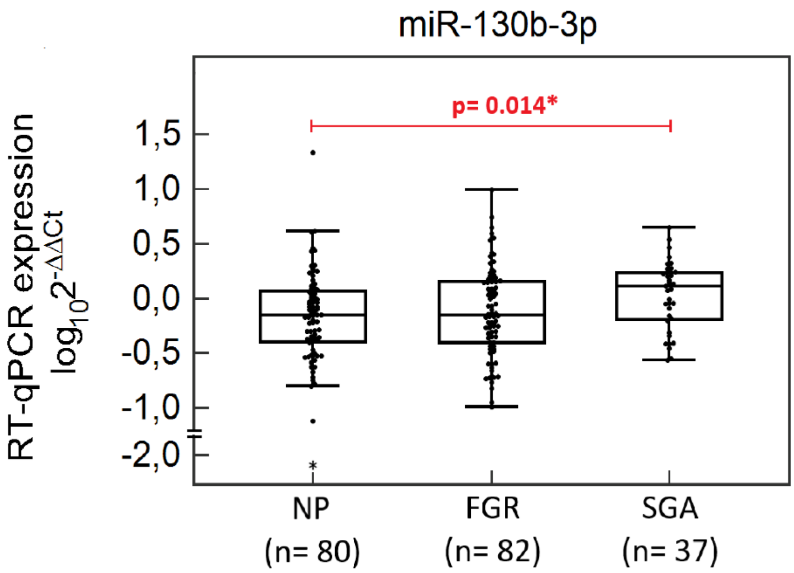

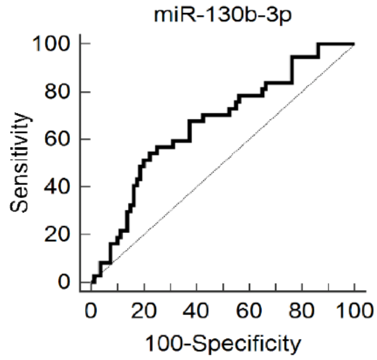

Area under the ROC curve (AUC)	0.665
Standard Error	0.0540
95% Confidence interval	0.572 to 0.750
Significance level P (Area=0.5)	0.0022

Estimated sensitivity at fixed specificity			
Specificity	Sensitivity	95% CI	Criterion
90.00	18.92	2.70 to 45.95	>1.856532

Criterion	Sensitivity	95% CI	Specificity	95% CI	+LR	95% CI	-LR	95% CI
>1.14816	56.76	39.5 - 72.9	75.00	64.1 - 84.0	2.27	1.4 - 3.6	0.58	0.4 - 0.9

I

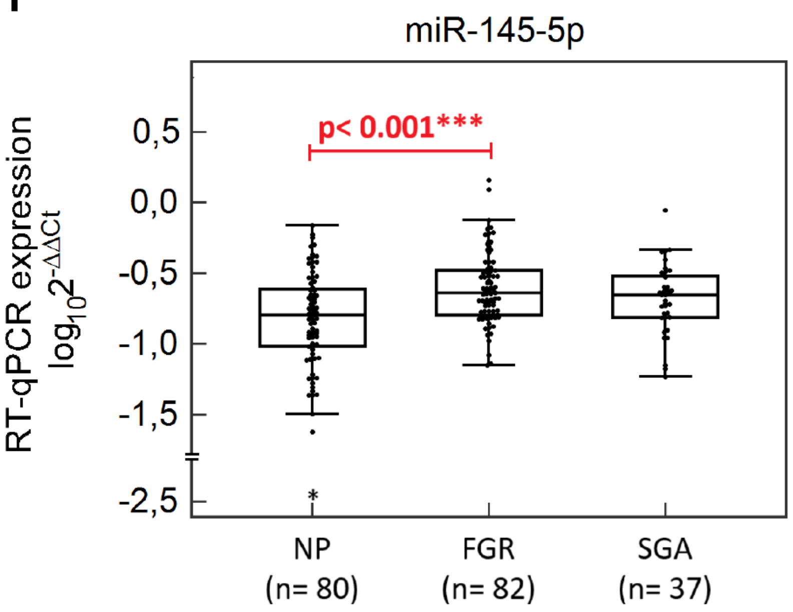

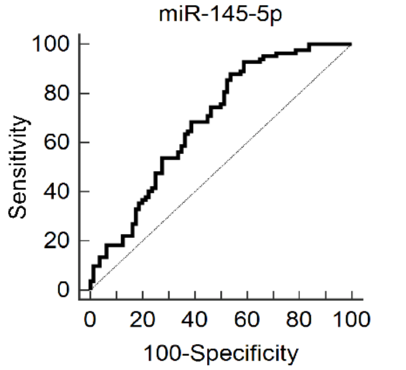

Area under the ROC curve (AUC)	0.687
Standard Error	0.0419
95% Confidence interval	0.610 to 0.758
Significance level P (Area=0.5)	<0.0001

Estimated sensitivity at fixed specificity			
Specificity	Sensitivity	95% CI	Criterion
90.00	18.29	8.54 to 30.49	>0.403208

Criterion	Sensitivity	95% CI	Specificity	95% CI	+LR	95% CI	-LR	95% CI
>0.149246	87.80	78.7 - 94.0	46.25	35.0 - 57.8	1.63	1.3 - 2.0	0.26	0.1 - 0.5

J

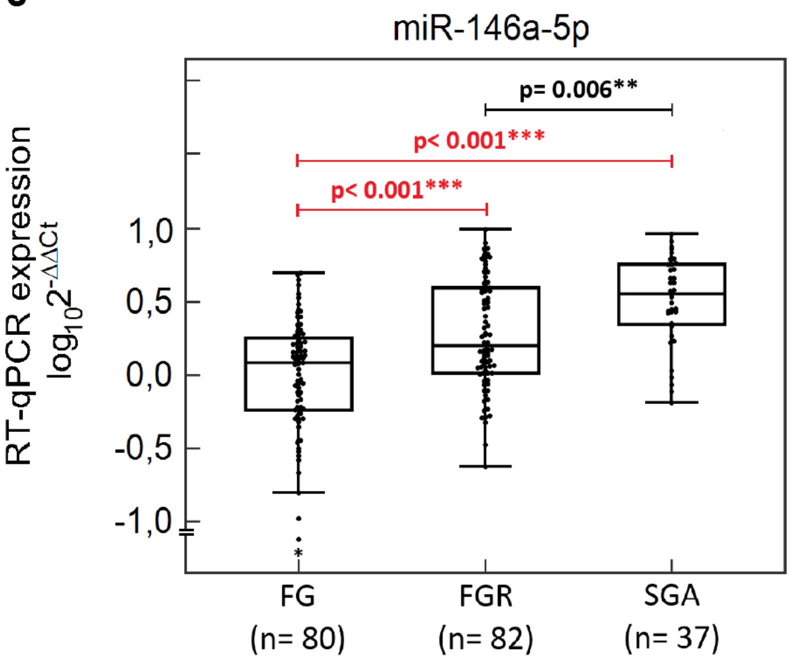

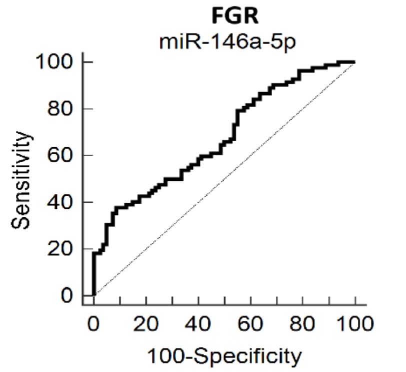

Area under the ROC curve (AUC)	0.676
Standard Error	0.0417
95% Confidence interval	0.598 to 0.747
Significance level P (Area=0.5)	<0.0001

Estimated sensitivity at fixed specificity			
Specificity	Sensitivity	95% CI	Criterion
90.00	37.80	24.39 to 51.22	>2.744534

Criterion	Sensitivity	95% CI	Specificity	95% CI	+LR	95% CI	-LR	95% CI
>2.820447	37.80	27.3 - 49.2	91.25	82.8 - 96.4	4.32	2.0 - 9.2	0.68	0.6 - 0.8

M

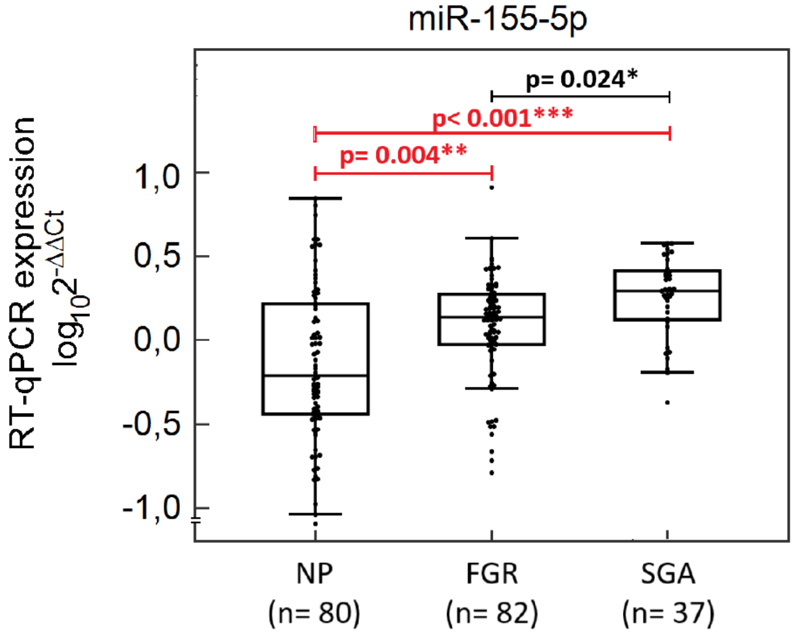

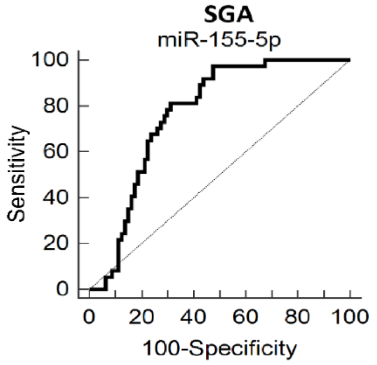

Area under the ROC curve (AUC)	0.767
Standard Error	0.0432
95% Confidence interval	0.680 to 0.840
Significance level P (Area=0.5)	<0.0001

Estimated sensitivity at fixed specificity			
Specificity	Sensitivity	95% CI	Criterion
90.00	8.11	0.00 to 43.24	>3.615596

Criterion	Sensitivity	95% CI	Specificity	95% CI	+LR	95% CI	-LR	95% CI
>1.103463	81.08	64.8 - 92.0	68.75	57.4 - 78.7	2.59	1.8 - 3.7	0.28	0.1 - 0.5

N

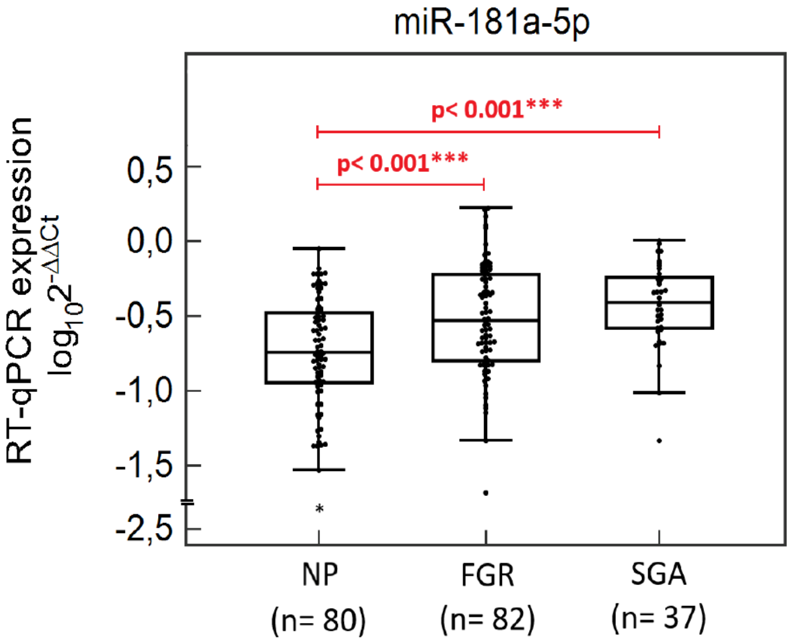

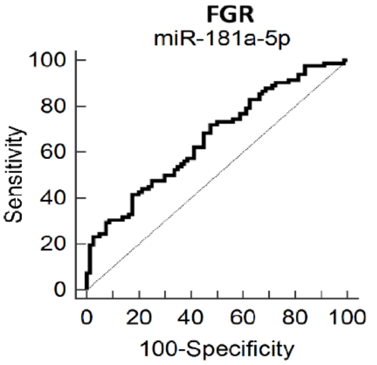

Area under the ROC curve (AUC)	0.663
Standard Error	0.0423
95% Confidence interval	0.584 to 0.735
Significance level P (Area=0.5)	0.0001

Estimated sensitivity at fixed specificity			
Specificity	Sensitivity	95% CI	Criterion
90.00	30.49	19.51 to 44.25	>0.521629

Criterion	Sensitivity	95% CI	Specificity	95% CI	+LR	95% CI	-LR	95% CI
>0.183648	71.95	60.9 - 81.3	52.50	41.0 - 63.8	1.51	1.2 - 2.0	0.53	0.4 - 0.8

O

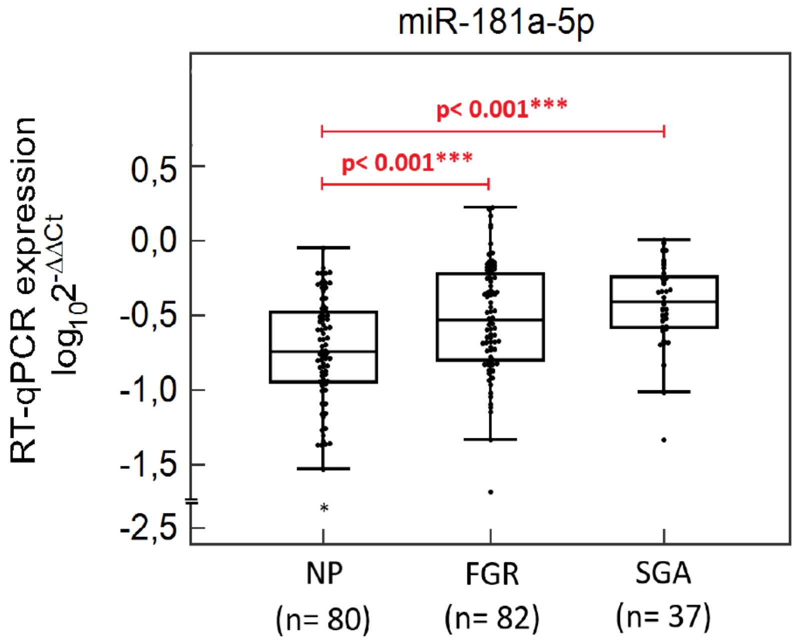

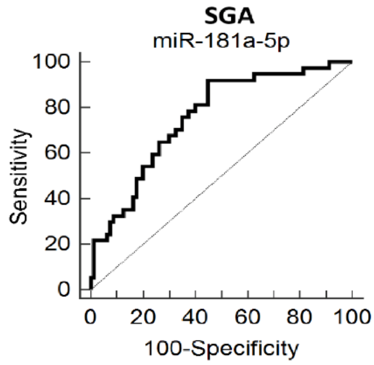

Area under the ROC curve (AUC)	0.757
Standard Error	0.0467
95% Confidence interval	0.669 to 0.831
Significance level P (Area=0.5)	<0.0001

Estimated sensitivity at fixed specificity			
Specificity	Sensitivity	95% CI	Criterion
90.00	32.43	16.22 to 56.76	>0.521629

Criterion	Sensitivity	95% CI	Specificity	95% CI	+LR	95% CI	-LR	95% CI
>0.201448	91.89	78.1 - 98.3	55.00	43.5 - 66.2	2.04	1.6 - 2.6	0.15	0.05 - 0.4

P

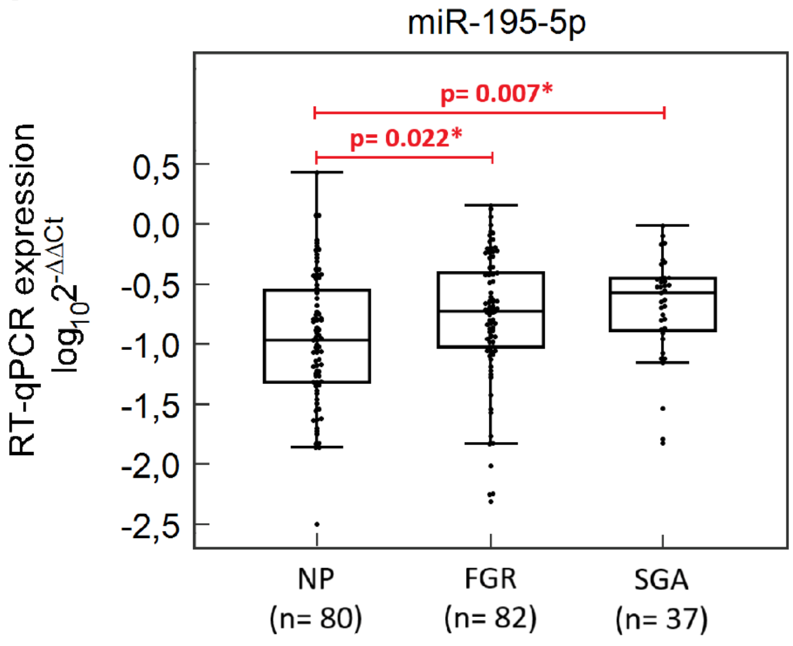

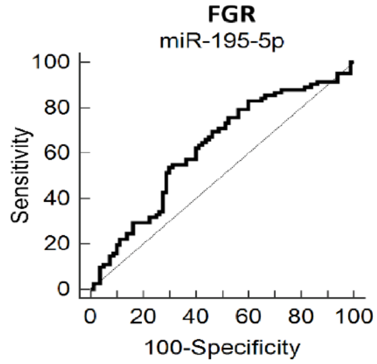

Area under the ROC curve (AUC)	0.623
Standard Error	0.0444
95% Confidence interval	0.544 to 0.698
Significance level P (Area=0.5)	0.0056

Estimated sensitivity at fixed specificity			
Specificity	Sensitivity	95% CI	Criterion
90.00	19.51	7.32 to 32.93	>0.553901

Criterion	Sensitivity	95% CI	Specificity	95% CI	+LR	95% CI	-LR	95% CI
>0.176527	53.66	42.3 - 64.7	70.00	58.7 - 79.7	1.79	1.2 - 2.6	0.66	0.5 - 0.9

Q

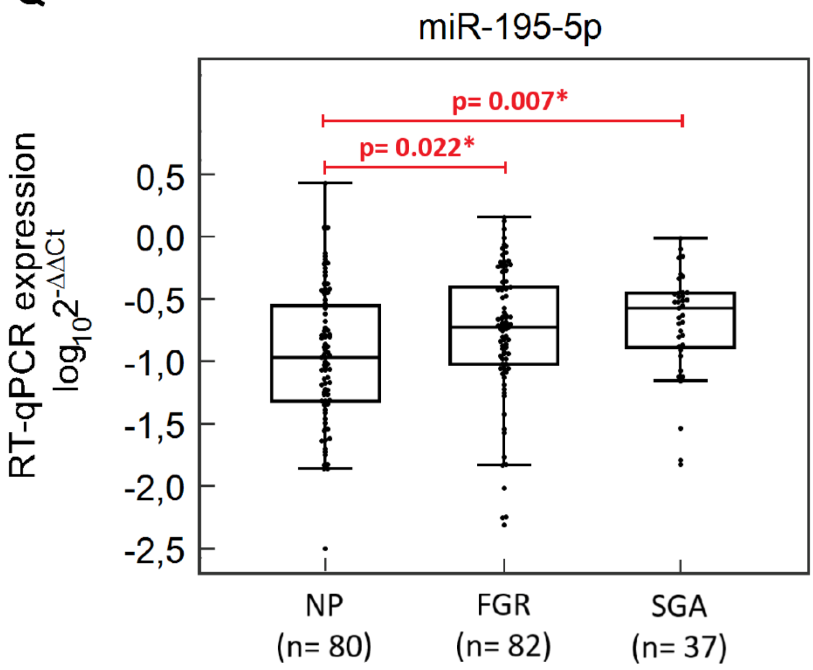

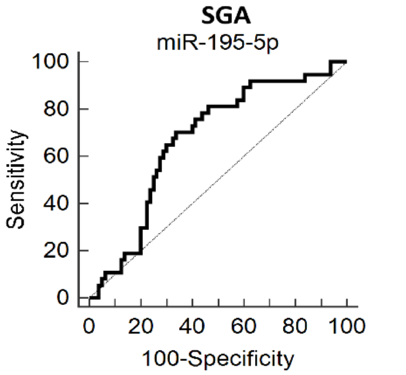

Area under the ROC curve (AUC)	0.672
Standard Error	0.0522
95% Confidence interval	0.579 to 0.756
Significance level P (Area=0.5)	0.0010

Estimated sensitivity at fixed specificity			
Specificity	Sensitivity	95% CI	Criterion
90.00	10.81	2.70 to 29.73	>0.553901

Criterion	Sensitivity	95% CI	Specificity	95% CI	+LR	95% CI	-LR	95% CI
>0.16052	70.27	53.0 - 84.1	66.25	54.8 - 76.4	2.08	1.4 - 3.0	0.45	0.3 - 0.8

R

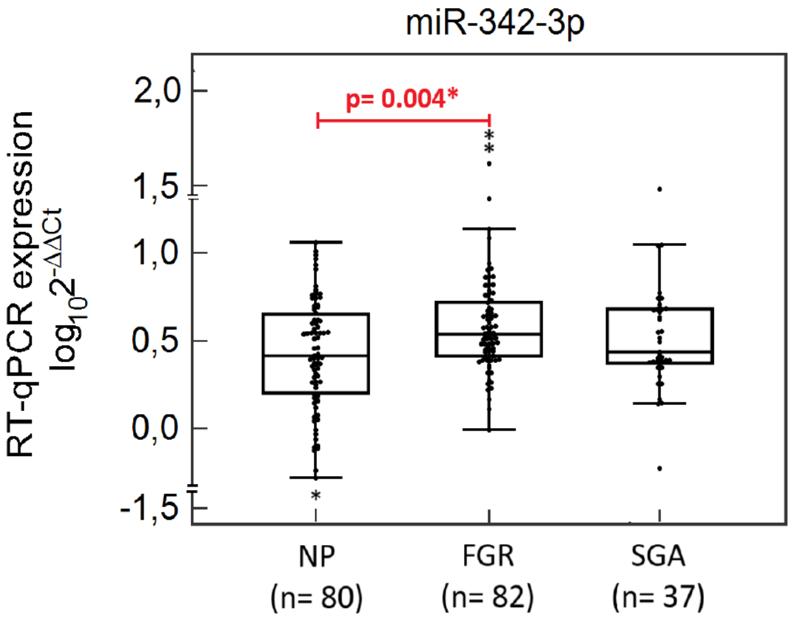

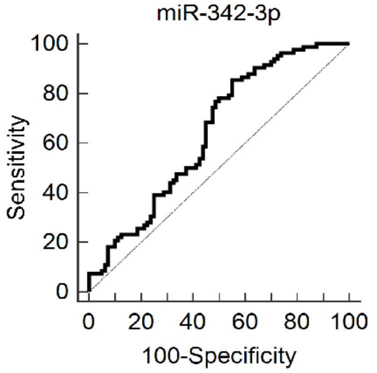

Area under the ROC curve (AUC)	0.642
Standard Error	0.0438
95% Confidence interval	0.563 to 0.716
Significance level P (Area=0.5)	0.0012

Estimated sensitivity at fixed specificity			
Specificity	Sensitivity	95% CI	Criterion
90.00	20.73	7.32 to 31.71	>5.904859

Criterion	Sensitivity	95% CI	Specificity	95% CI	+LR	95% CI	-LR	95% CI
>2.333226	85.37	75.8 - 92.2	45.00	33.8 - 56.5	1.55	1.2 - 1.9	0.33	0.2 - 0.6

S

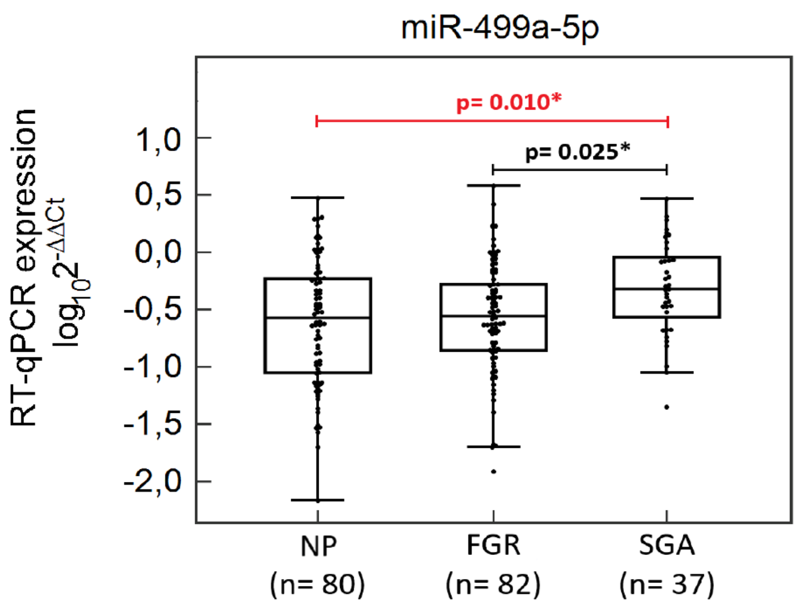

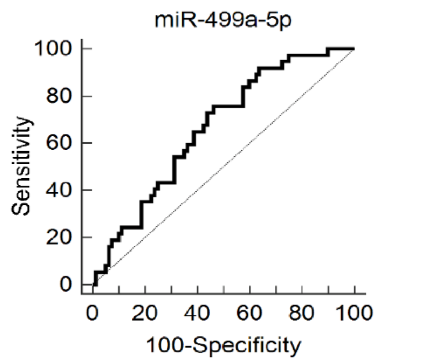

Area under the ROC curve (AUC)	0.663
Standard Error	0.0514
95% Confidence interval	0.570 to 0.748
Significance level P (Area=0.5)	0.0015

Estimated sensitivity at fixed specificity			
Specificity	Sensitivity	95% CI	Criterion
90.00	21.62	5.41 to 39.31	>1.191424

Criterion	Sensitivity	95% CI	Specificity	95% CI	+LR	95% CI	-LR	95% CI
>0.299081	75.68	58.8 - 88.2	53.75	42.2 - 65.0	1.64	1.2 - 2.2	0.45	0.2 - 0.8

T

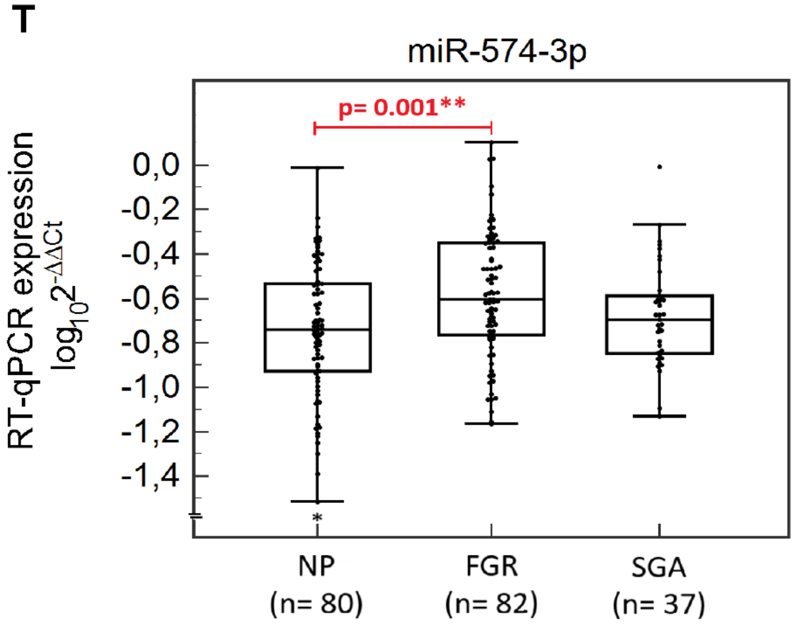

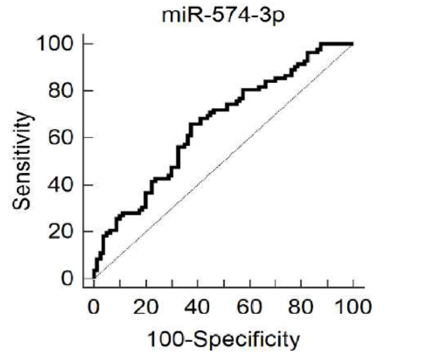

Area under the ROC curve (AUC)	0.655
Standard Error	0.0428
95% Confidence interval	0.576 to 0.727
Significance level P (Area=0.5)	0.0003

Estimated sensitivity at fixed specificity			
Specificity	Sensitivity	95% CI	Criterion
90.00	26.83	15.85 to 39.16	>0.42623

Criterion	Sensitivity	95% CI	Specificity	95% CI	+LR	95% CI	-LR	95% CI
>0.202286	65.85	54.6 - 76.0	62.50	51.0 - 73.1	1.76	1.3 - 2.4	0.55	0.4 - 0.8

Figure S1: Gene expression of cardiovascular disease-associated microRNAs in peripheral blood leukocytes in early stages of gestation—comparison between appropriate-for-gestational-age (AGA) fetuses, small-for-gestational-age (SGA) fetuses, and fetal growth-restricted (FGR) fetuses; statistical significant data after Benjamini–Hochberg correction (results after the Benjamini-Hochberg correction are marked by * for α=0.05, ** for α=0.01, and *** for α=0.001).

NP, normal pregnancies (appropriate-for-gestational-age fetuses); FGR, fetal growth restricted fetuses; SGA, small-for-gestational-age fetuses
