# Supplementary material for: First-Trimester Screening for Fetal Growth Restriction and Small-for-Gestational-Age Pregnancies without Preeclampsia Using Cardiovascular Disease-Associated MicroRNA Biomarkers
Source: Biomedicines. 2022 Mar 19;10(3):718. doi: 10.3390/biomedicines10030718 (PMC8945808; doi:10.3390/biomedicines10030718)
Supplement: Supplementary file 1 [file biomedicines-10-00718-s001.zip › Supplementary Figure S2.pdf]

Supplementary Figure S2.

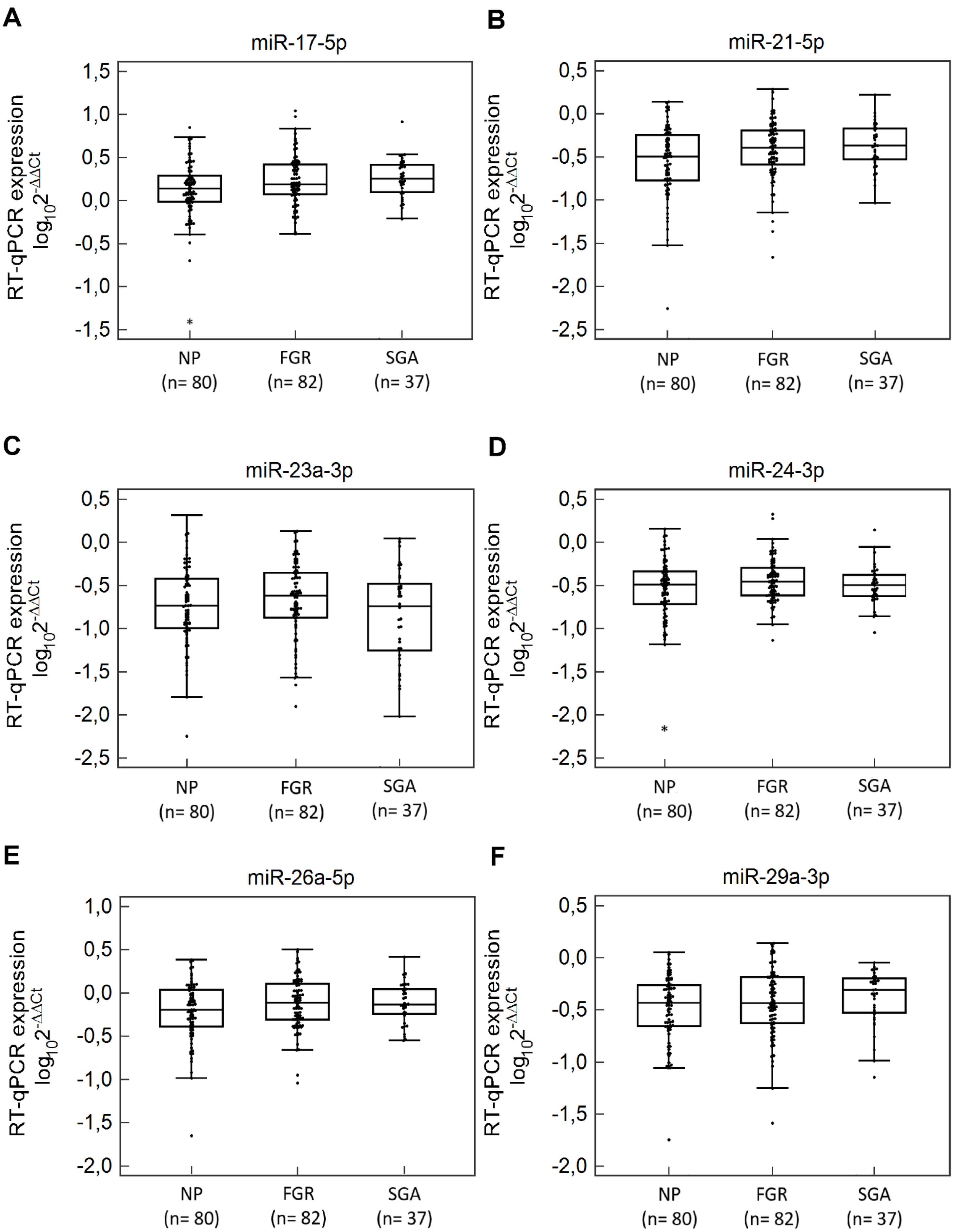

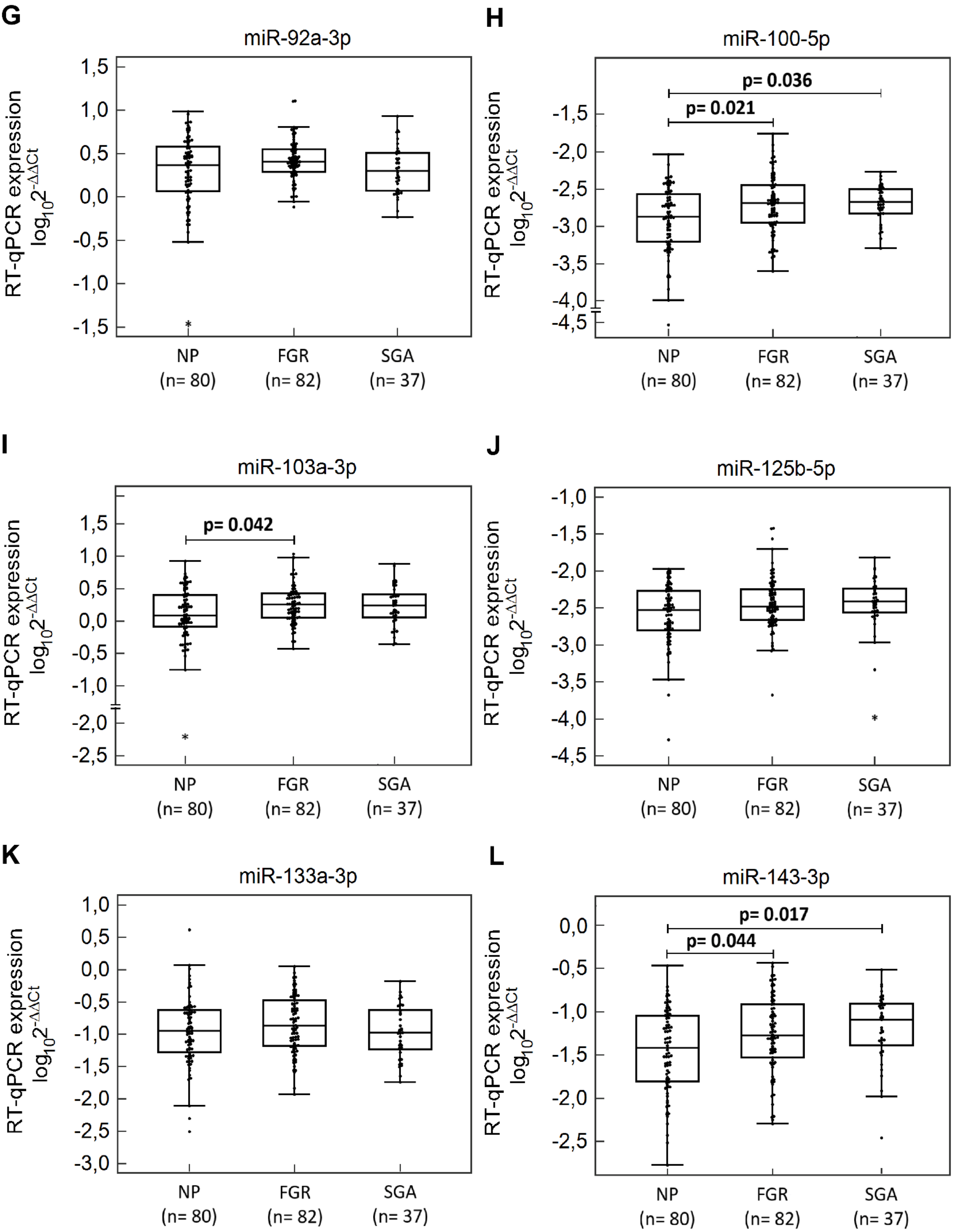
G
miR-92a-3p
RT-qPCR expression
$\log_{10}2^{-\Delta\Delta Ct}$
1,5
1,0
0,5
0,0
-0,5
-1,0
-1,5
NP
(n= 80)
FGR
(n= 82)
SGA
(n= 37)
H
miR-100-5p
p= 0.036
p= 0.021
-1,5
-2,0
-2,5
-3,0
-3,5
-4,0
-4,5
I
miR-103a-3p
p= 0.042
1,5
1,0
0,5
0,0
-0,5
-1,0
-2,0
-2,5
J
miR-125b-5p
-1,0
-1,5
-2,0
-2,5
-3,0
-3,5
-4,0
-4,5
K
miR-133a-3p
1,0
0,5
0,0
-0,5
-1,0
-1,5
-2,0
-2,5
-3,0
L
miR-143-3p
p= 0.017
p= 0.044
0,0
-0,5
-1,0
-1,5
-2,0
-2,5

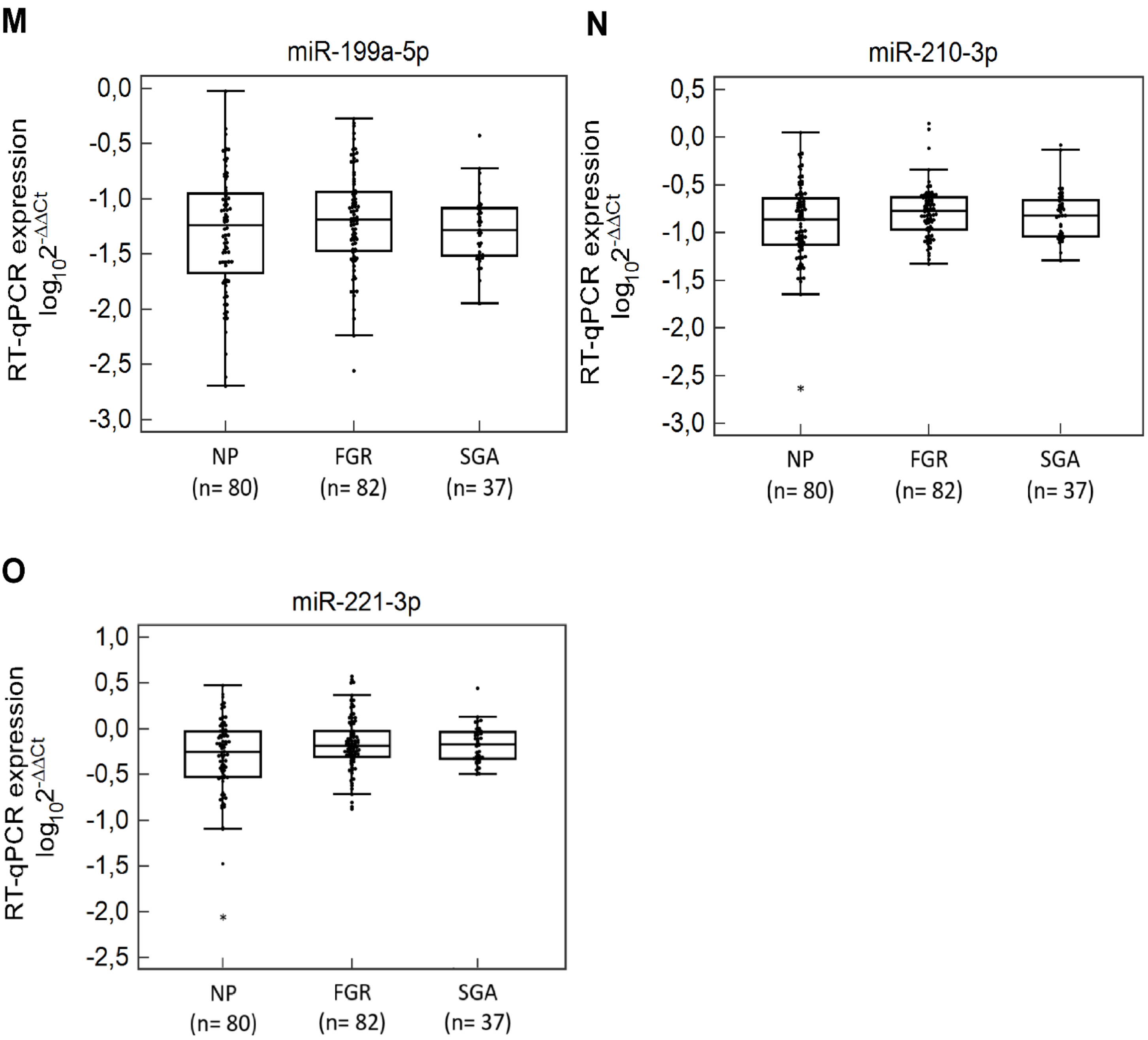

Figure S2: Gene expression of cardiovascular disease-associated microRNAs in peripheral blood leukocytes in early stages of gestation–comparison between appropriate-for-gestational-age (AGA) fetuses, small-for-gestational-age (SGA) fetuses, and fetal growth-restricted (FGR) fetuses—statistical non-significant data after Benjamini–Hochberg correction.
NP, normal pregnancies (appropriate-for-gestational-age fetuses); FGR, fetal growth restricted fetuses; SGA, small-for-gestational-age fetuses
